# Use of complete blood count for predicting preterm birth in asymptomatic pregnant women: A propensity score‐matched analysis

**DOI:** 10.1002/jcla.23313

**Published:** 2020-03-27

**Authors:** Mei Ma, Mei Zhu, Bimin Zhuo, Li Li, Honglei Chen, Libo Xu, Zhihui Wu, Feng Cheng, Liangpu Xu, Jianying Yan

**Affiliations:** ^1^ Department of Laboratory Medicine Fujian Provincial Maternity and Children’s Hospital Affiliated Hospital of Fujian Medical University Fuzhou China; ^2^ Department of Neurosurgery The Affiliated People's Hospital of Fujian University of Traditional Chinese Medicine Fuzhou China; ^3^ Department of Obstetrics and Gynecology Fujian Provincial Maternity and Children's Hospital Affiliated Hospital of Fujian Medical University Fuzhou China; ^4^ Computer Technology Department Fujian Provincial Maternity and Children’s Hospital Affiliated Hospital of Fujian Medical University Fuzhou China

**Keywords:** complete blood count, Inflammation, neutrophil‐to‐lymphocyte ratio, pregnancy, preterm birth, preterm delivery, preterm labor, propensity score‐matched analysis

## Abstract

**Background:**

Accurate prediction of preterm birth (PTB) is still difficult, mostly because of the multifactorial etiology of PTB. Previous studies have been mostly focused on the prediction of PTB in symptomatic women or those presenting with threatened preterm labor. We aimed to study whether complete blood count (CBC) parameters at 20‐30 weeks of pregnancy can predict asymptomatic PTB.

**Methods:**

In this retrospective case‐control study, the preterm and term delivery groups were matched by propensity score‐matched (PSM) analysis. Baseline data and the CBC parameters examined at 20‐30 weeks of gestation were recorded.

**Results:**

The combined marker of neutrophil‐to‐lymphocyte ratio (NLR), hemoglobin (HGB), and platelet distribution width (PDW) accurately predicts PTB at a cutoff value of 0.25, with sensitivity and specificity of 88.6% and 40.5% and negative and positive predictive value of 97.9% and 10.2%, respectively.

**Conclusion:**

The combined marker of CBC parameters can supplement other markers to predict PTB about 10 weeks in advance. This combined marker had a very high negative predictive value for PTB. Therefore, in subjects with normal combined marker value, further screening tests for PTB may be eliminated unless clinical suspicion is high.

## 1 INTRODUCTION

Preterm birth (PTB), defined as birth before 37 complete weeks of gestation, is a very serious obstetric problem worldwide. It affects 5%‐18% of all deliveries in the world and represents an enormous health care burden.^1^, ^2^ It is the largest direct cause of both neonatal and child (under the age of 5 years) mortality.^3^ In 2015, preterm birth complication (1.055 million) was the leading cause of death among the 5.9 million children under 5 years old worldwide.^4^ In addition to its contribution to mortality, there is increasing evidence that PTB infants are at increased risk of developing some diseases such as respiratory distress syndrome, jaundice, temperature instability, feeding difficulties, developmental disabilities, and neurologic impairments.^5^, ^6^, ^7^ Furthermore, the increased risk of some adult diseases, such as cardiovascular diseases, type 2 diabetes, stroke, asthma, and some behavior and psychiatric disorders, has also been linked to PTB.^8^, ^9^

The premature birth after spontaneous delivery and preterm premature rupture of membranes(PPROM) are collectively referred to as spontaneous PTB, and those after induction or cesarean labor (including maternal or fetal complications requiring delivery) are called iatrogenic PTB.^10^ Accumulating evidence suggests that PTB is a complex syndrome resulting from the interplay of genetic, environmental, and lifestyle risk factors. It is associated with microbial‐induced inflammation, decidual hemorrhage and vascular disease, decidual senescence, disruption of maternal‐fetal tolerance, decline in progesterone action, uterine over‐distension, stress, and others.^11^ Research so far has shown that accurate predictions of PTB, especially the use of a single biomarker to predict PTB, are still difficult, because of the multifactorial etiology of PTB.^6^

Predicting PTB is still mostly dependent on subjective clinical experience in most countries. Such approach may increase unnecessary hospital admissions as well as unnecessary but potentially harmful interventions such as the use of steroids for fetal lung maturation and of tocolysis.^12^, ^13^ To improve the accuracy of threatened PTB diagnosis in symptomatic women, two different methods have been suggested: transvaginal ultrasound cervical length measurement and measurement of fetal fibronectin (fFN)/placental alpha microglobulin‐1(PAMG1)/phosphorylated insulin‐like growth factor binding protein‐1 (phIGFBP 1) in the cervicovaginal fluid(CVF).^14^

Since their introduction nearly 28 years ago, cervical length screening and fFN levels have been widely used to predict PTB.^15^, ^16^ In recent years, however, some studies have shown low predictive accuracy of these two tests. A systematic review and meta‐analysis found that fFN should not be used as a screening test for asymptomatic pregnant women. For high‐risk asymptomatic pregnant women, and especially for women with multiple pregnancies, the predictive value of the test was also too low for clinical practice.^17^ Another review and meta‐analysis did suggest that although management based on fFN results may reduce PTB, the evidence was found to be of low quality.^18^ A prospective observational cohort study with 9410 nulliparous women of singleton pregnancies shows that quantitative vaginal fFN and serial transvaginal ultrasound cervical length had limited predictive value for PTB. The study does not support routine application of these tests in the clinic for those pregnancies.^19^ Cervical phIGFBP‐1 has potential utility in identifying patients who had one episode of preterm labor but remained undelivered within 48 hours, based on a systematic review and meta‐analysis.^20^ However, its overall predictive ability to identify symptomatic and asymptomatic women at risk of PTB is limited.^20^ While the sensitivities of PAMG1 and phIGFBP1 were comparable in predicting spontaneous PTB within 7 days, the specificity of PAMG1 was significantly higher than phIGFBP1.^21^ Another limitation of these studies is that they focused mostly on the prediction of PTB within 7 or 14 days in women presenting with threatened preterm labor. More recently, whole genome sequencing, whole exome sequencing, and cell‐free RNA (cfRNA) have been used to identify genes or RNA that may predict preterm delivery.^22^, ^23^, ^24^ These new approaches hold promise in identifying specific biomarkers for spontaneous PTB. Validation in larger and blinded clinical trials in diverse ethnicities and different populations is needed however before they gain widespread clinical application.^22^, ^23^

It is well established that one highly significant risk factor for PTB is infection and inflammation.^24^ One of the most simple, economic, and routine clinical tests during pregnancy is the complete blood count (CBC). CBC and its derived parameters, including neutrophil‐to‐lymphocyte ratio (NLR) and platelet‐to‐lymphocyte ratio (PLR), have been recognized as inflammatory markers for low‐grade inflammatory diseases in recent years.^25^, ^26^, ^27^ There is accruing evidence that NLR can have a significant association with inflammatory response observed in a variety of disorders, including cerebrovascular and heart diseases,^28^, ^29^, ^30^ and can improve outcome prediction of such diseases when added to traditional risk scores and tools.^31^ The present study examines whether CBC variables including inflammation‐related indicators at 20‐30 weeks of pregnancy can predict asymptomatic preterm delivery.

## 2 MATERIALS AND METHODS

### 2.1 Study design

This was a retrospective case‐control study. We reviewed the clinical data of all (15 387 total) pregnant women for preterm deliveries and term deliveries between January and December 2017 at Fujian Provincial Maternity and Children's Hospital, China. The preterm delivery group and term delivery group were matched by PSM analysis. The study was approved by the Medical Ethics Committee of the hospital (2019 No. 164).

### 2.2 Study participants

Pregnant women who delivered prematurely at 28‐36 weeks of gestation were included in the study group, and those who delivered at 37‐40 weeks of gestation were included in our control group. The inclusion criteria were singleton pregnancy, no uterine and placental abnormalities, no infection or chronic inflammatory diseases, no fever, no heart disease, no cervical insufficiency, no history of cervical cerclage, and no history of taking medications affecting platelet count. Exclusion criteria were gestational diabetes mellitus, hypertensive disorder complicating pregnancy, pre‐eclampsia, chorioamnionitis, severe anemia, thyroid disease, intrauterine growth retardation, and recurrent miscarriage. These pregnant women received CBC tests during 20‐30 weeks of gestation.

### 2.3 Clinical and laboratory data collection

Medical records were reviewed to collect baseline data including the maternal age, height and body mass index (BMI) before pregnancy, gravidity, parity, the gestational age at delivery, gestational age at CBC assessment, the delivery weight, and PPROM. CBC variables 20‐30 weeks of gestation were also recorded (CBC obtained during active childbirth was excluded). Sysmex‐SN3000 blood cell counter was used as a blood cell counting machine. CBC parameters used in the study were as follows: WBC, white blood cell; Neu#, neutrophil count; Lym#, lymphocyte count; Mon#, monocyte count; HGB, hemoglobin; HCT, hematocrit; RDW, red cell distribution width; PLT, platelet count; MPV, mean platelet volume; PCT, plateletcrit; PDW, platelet distribution width; NLR, neutrophil‐to‐lymphocyte ratio; LMR, lymphocyte‐to‐monocyte ratio; PLR, platelet‐to‐lymphocyte ratio.

### 2.4 Statistical analysis

Propensity score‐matched analysis was performed using R programming language (San Francisco, CA), according to the ratio of 1:2 cases. The premature deliveries and full‐term deliveries were matched in age, BMI, whether the pregnant woman had PPROM, and gestational age the pregnant women received CBC examinations. The nearest neighbor matching algorithm was used in the PMS analysis. The statistical software SPSS for Windows version 20.0 (SPSS Inc) was used to analyze whether there was a significant difference in CBC parameters between the two groups. The distributions of the data were determined by the Kolmogorov‐Smirnov test, and the results of the variables were expressed as means ± standard deviation or median (minimum—maximum). Independent sample Student's t test or Mann‐Whitney *U* test was performed to compare continuous numeric variables. The receiver operating characteristic (ROC) curve for each CBC parameter was plotted using MedCalc v.14.12.0 (MedCalc Software bvba). The best cutoff points for CBC parameters to discriminate preterm deliveries and term deliveries were evaluated by the area under the curve (AUC) of the ROC. The ability of each CBC parameter to identify PTB was assessed by comparing the AUC of the ROC. The combined marker value for discriminating PTB from term delivery was calculated by binary logistic regression. Two‐sided *P*‐values <.05 were considered statistically significant.

## 3 RESULTS

### 3.1 Study participants and baseline characteristics

A total of 1634 of the pregnant women fulfilled the inclusion criteria (Table 1). Of these pregnant women, 105 were diagnosed preterm labor, and 1529 were diagnosed for term labor. As presented in Table 1, 68.4% of the premature delivery group had PPROM, while only 29.2% of the 1529 full‐term deliveries had PPROM before labor. The incidence of PPROM was statistically significant between the two groups (preterm and term delivery group *P* < .001). In addition, clinical features such as BMI, age, and gestational age at assessment may have an impact on the results of CBC. After adjusting for these four clinical features with PSM analysis, a total of 105 and 210 pregnant women were retained in the study group and control group, respectively. The incidence of PPROM after the adjustment was not significantly different between the groups (*P* = .943). Delivery weight and gestational age at delivery were significantly lower in the preterm group than in the term group before and after PSM analysis, and the differences were significant (*P* < .001 for both). Gestational age at assessment of CBC parameters was not significantly different between the groups (about 24.8 weeks for both, *P* = .871).

**TABLE 1:** Demographic and obstetric characteristics of the preterm delivery and control group before and after PSM analysis

	Pre‐PSM			Post‐PSM		
	Preterm delivery (n = 105)	Term delivery (n = 1529)	*P*‐value	Preterm delivery (n = 105)	Term delivery (n = 210)	*P*‐value
Maternal age (y)	29.93 ± 4.039	29.59 ± 3.823	.381	29.93 ± 4.039	30.07 ± 4.201	.781
Pre‐pregnancy IBM (kg/m^2^)	20.17 ± 2.116	20.59 ± 8.425	.619	20.17 ± 2.116	20.26 ± 2.316	.760
Gravidity (number)	1.71 ± 0.917	1.76 ± 0.905	.592	1.71 ± 0.917	1.90 ± 0.988	.100
Parity (number)	0.33 ± 0.494	0.37 ± 0.527	.502	0.33 ± 0.494	0.45 ± 0.611	.075
Gestational age at assessment (wk)	24.75 ± 1.663	24.8 ± 1.608	.779	24.75 ± 1.663	24.8 ± 1.487	.871
Gestational age at delivery(weeks)	35.2 ± 1.477	39.25 ± 1.085	.000	35.2 ± 1.477	39.06 ± 1.040	.000*
Assessment to delivery interval (d)	71.1 ± 15.0	99.2 ± 13.5	.000	70.2 ± 15.2	97.0 ± 12.4	.000*
Delivery weight (g)	2573.1 ± 442.59	3338.9 ± 362.95	.000	2573.1 ± 442.59	3344.9 ± 366.96	.000*
PPROM	64.8% (68/105)	29.2% (447/1529)	.000	64.8% (68/105)	65.2% (137/210)	.934

Abbreviation: PPROM, preterm premature rupture of membranes.

* *P* < .05 was accepted as significant.

### 3.2 CBC parameters of the two groups

The CBC parameters of the two groups before and after PSM analysis are shown in Table 2. As indicated in the table, NLR, MLR, PLR, PDW, and lymphocytes were statistically different between the two groups before PSM analysis. After PSM analysis, the statistical differences between the first four indicators (NLR, MLR, PLR, and PDW) were more statistically significant (*P*‐value is smaller), in addition, HGB and MPV, HCT also reached statistical differences between the two groups after PSM analysis. The mean values of NLR, PDW, MPV, HGB, HCT, PLR were significantly higher (*P* < .001, *P* = .005, .037, .010, .017, respectively), and LMR and lymphocytes were significantly lower (*P* = .003, .016, .012, respectively) in preterm delivery group after PSM analysis.

**TABLE 2:** Comparison of CBC parameters between the preterm delivery and control groups

	Pre‐PSM			Post‐PSM		
	Preterm delivery (n = 105)	Term delivery (n = 1529)	*P*‐value	Preterm delivery (n = 105)	Term delivery (n = 210)	*P*‐value
WBC count (103/mm^3^)	10.18 ± 2.54	9.94 ± 2.13	.350	10.18 ± 2.54	9.90 ± 1.95	.317
Neu# (103/mm^3^)	7.58 ± 2.13	7.27 ± 1.82	.098	7.58 ± 2.13	7.19 ± 1.66	.106
Lym# (103/mm^3^)	1.80 ± 0.50	1.89 ± 0.44	.029*	1.80 ± 0.50	1.93 ± 0.42	.012*
Mon# (103/mm^3^)	0.65 ± 0.21	0.63 ± 0.17	.180	0.65 ± 0.21	0.62 ± 0.16	.195
HGB (g/L)	115.09 ± 9.42	113.52 ± 8.67	.075	115.09 ± 9.42	112.31 ± 8.74	.010 *
HCT (%)	33.79 ± 2.51	33.33 ± 2.34	.052	33.79 ± 2.51	33.10 ± 2.34	.017*
RDW (%)	13.28 ± 1.85	13.09 ± 0.80	.292	13.28 ± 1.85	13.13 ± 0.80	.415
PLT (103/mm^3^)	217.92 ± 51.32	219.75 ± 46.30	.697	217.92 ± 51.32	219.56 ± 46.81	.778
MPV (fL)	10.33 ± 1.00	10.18 ± 0.87	.082	10.33 ± 1.00	10.10 ± 0.90	.037 *
PCT (%)	0.22 ± 0.05	0.22 ± 0.04	.611	0.22 ± 0.05	0.22 ± 0.04	.971
PDW (%)	12.03 ± 2.49	11.53 ± 2.01	.046*	12.03 ± 2.49	11.31 ± 1.95	.005*
NLR	4.42 ± 1.41	3.99 ± 1.21	.000*	4.42 ± 1.41	3.86 ± 1.09	.000*
LMR	2.94 ± 0.97	3.19 ± 0.91	.007*	2.94 ± 0.97	3.28 ± 0.98	.003*
PLR	128.75 ± 39.58	120.73 ± 33.30	.045*	128.75 ± 39.58	117.96 ± 31.32	.016*

Abbreviations: HCT, hematocrit; HGB, hemoglobin; LMR, lymphocyte‐to‐monocyte ratio; Lym#, lymphocyte count; Mon#, monocyte count; MPV, mean platelet volume; Neu#,neutrophil count; NLR, neutrophil‐to‐lymphocyte ratio; PCT, plateletcrit; PDW, platelet distribution width; PLR, platelet‐to‐lymphocyte ratio; PLT, platelet count; RDW, red cell distribution width; WBC, white blood cell.

* *P* < .05 was accepted as significant.

### 3.3 ROC and combined marker

**FIGURE 1:**
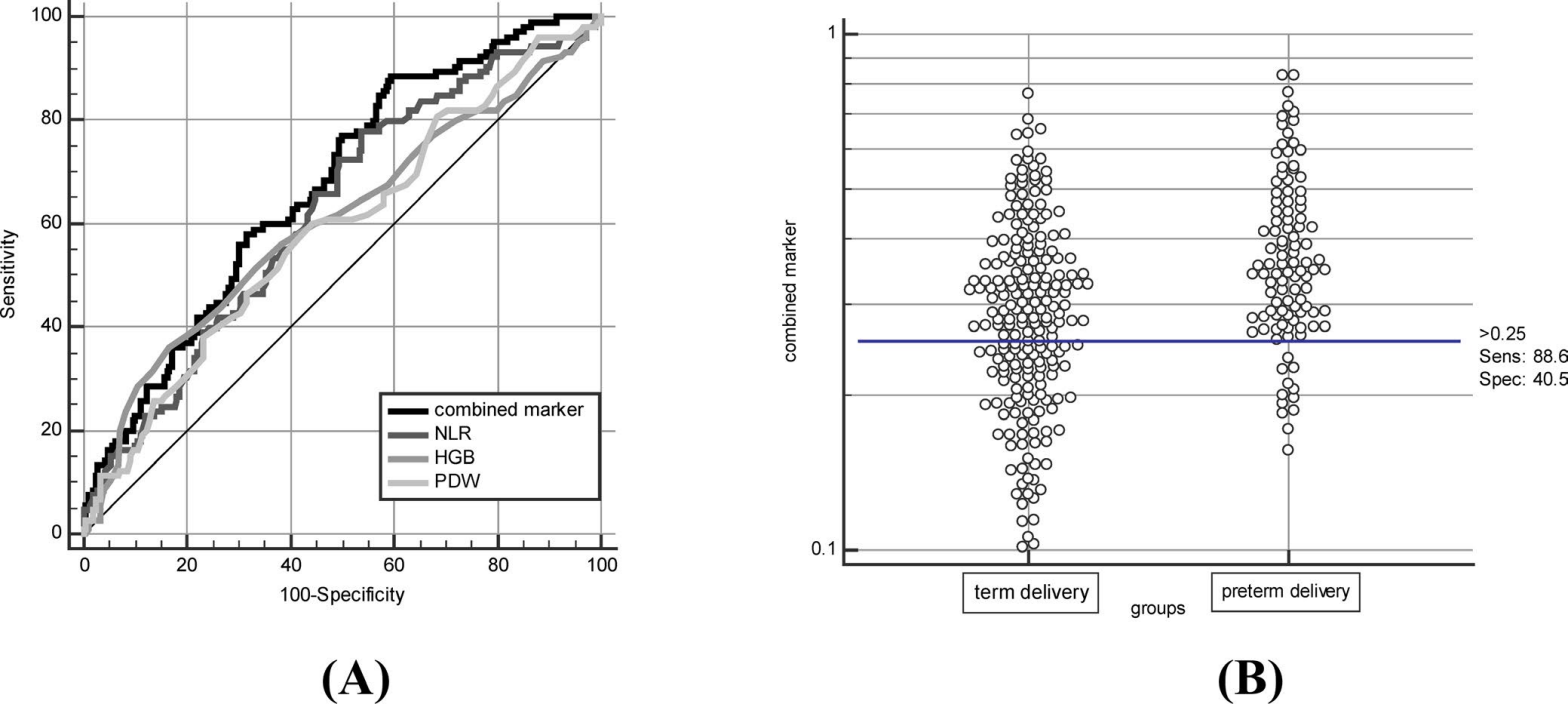
(A) Receiver operating characteristic (ROC) curve for NLR, HGB, PDW, and combined marker for the diagnosis of PTB. (B) Interactive dot diagram for the combined marker

We also analyzed the ROC to compare the diagnostic usefulness of CBC parameters (Figure 1 and Table 3). ROC analysis suggested that NLR, HGB, and PDW had the highest area under the curve (AUC = 0.625, 0.604, and 0.588, respectively.) among the parameters of leukocytes, erythrocytes, and platelets in predicting preterm delivery. In addition, the combined marker of NLR, HGB, and PDW can accurately predict PTB at a cutoff value of 0.25 (AUC s 0.672 [95% CI is 0.618‐0.724]), with sensitivity and specificity of 88.6% and 40.5% and negative and positive prediction value of 97.9% and 10.2%, respectively.

**TABLE 3:** Diagnostic sensitivity and specificity of CBC parameters in study subjects

	AUC (95% CI)	Sensitivity (%) (95% CI)	Specificity (%) (95% CI)	+LR (95% CI)	‐LR (95% CI)	PPV (%) (95% CI)	NPV (%) (95% CI)	Cutoff value
HGB	0.604 (0.548‐0.658)	36.2 (27.0‐46.1)	83.3 (77.6‐88.1)	2.17 (1.5‐3.2)	0.77 (0.7‐0.9)	14.2 (10.1‐19.8)	94.5 (93.6‐95.2)	>119
HCT	0.591 (0.538‐0.650)	51.4 (41.5‐61.3)	69.1 (62.3‐75.2)	1.66 (1.3‐2.2)	0.7 (0.6‐0.9)	11.3 (8.8‐14.3)	94.9 (93.7‐95.9)	>34.2
MPV	0.569 (0.512‐0.624)	61.0 (50.9‐70.3)	51.9 (44.9‐58.8)	1.27 (1.0‐1.6)	0.75 (0.6‐1.0)	8.8 (7.3‐10.7)	94.6 (93.0‐95.8)	>10
PDW	0.588 (0.531‐0.643)	60.0 (50.0‐69.4)	55.7 (48.7‐62.5)	1.35 (1.1‐1.7)	0.72 (0.6‐0.9)	9.4 (7.7‐11.4)	94.8 (93.3‐96.0)	>11.2
Lym	0.598 (0.541‐0.652)	68.6 (58.8‐77.3)	49.5 (42.6‐56.5)	1.36 (1.1‐1.6)	0.63 (0.5‐0.9)	9.4 (7.9‐11.1)	95.4 (93.8‐96.6)	≤1.95
PLR	0.575 (0.519‐0.630)	74.3 (64.8‐82.3)	41.4 (34.7‐48.4)	1.27 (1.1‐1.5)	0.62 (0.4‐0.9)	8.8 (7.6‐10.2)	95.5 (93.6‐96.8)	>108.17
LMR	0.615 (0.559‐0.669)	31.4 (22.7‐41.2)	87.1 (81.8‐91.4)	2.44 (1.6‐3.8)	0.79 (0.7‐0.9)	15.7 (10.6‐22.7)	94.3 (93.5‐95.0)	≤2.31
NLR	0.625 (0.569‐0.679)	78.1 (69.0‐85.6)	46.2 (39.3‐53.2)	1.45 (1.2‐1.7)	0.47 (0.3‐0.7)	10 (8.6‐11.5)	96.5 (94.9‐97.6)	>3.56
Combined marker	0.672 (0.618‐0.724)	88.6 (80.9‐94.0)	40.5 (33.8‐47.4)	1.49 (1.3‐1.7)	0.28 (0.2‐0.5)	10.2 (9.1‐11.5)	97.9 (96.4‐98.8)	>0.25

## 4 DISCUSSION

In this PSM study, we investigated the ability of simple, noninvasive, and economic CBC tests to predict spontaneous PTB. At gestational age of 20‐30 weeks (mean of gestational age is 24 weeks), the mean values of NLR, PDW, MPV, HGB, HCT, PLR were significantly higher, and LMR and lymphocytes were significantly lower in preterm delivery group than in the term delivery group. Another major finding is that NLR, HGB, and PDW in white blood cell classification, red blood cell‐related parameters, and platelet parameters had the greatest predictive value for PTB, respectively. Furthermore, the combined marker of the above three parameters could better predict spontaneous PTB than a single marker.

### 4.1 PTB and inflammatory factors

Despite the wide range of etiology, it is well accepted that PTB is strongly associated with infection and inflammation.^32^ Premature initiation was closely related to changes in inflammatory media and their signaling pathways. Previous studies have shown that inflammatory cytokines such as IL‐1,^33^ TNF‐alpha,^34^ IL‐6,^34^, ^35^ CRP,^35^ and IP‐10^35^ play an important role in the occurrence of PTB. NLR, a commonly available parameter that integrates information of the leukocyte differentials, has been proposed as a marker of systemic inflammation.^36^ A study of HKDAGLA et al^37^ showed that NLR was significantly higher in the preterm group than in the control group, which is consistent with our study. A key difference between our study and their previous investigation was the gestational age at assessment of CBC. Their blood routine examination time was after the onset of threatened PTB (31.1 ± 1.9 W in the preterm group). In our study, CBC obtained during active labor was not used. We used CBC values detected within 10 weeks prior to the hospitalization of the pregnant woman. (premature delivery group is 24.8 ± 1.7 W). Min‐A kim et al^38^ analyzed CBC for predicting PTB in symptomatic pregnant women at an average gestational age of 28.6 ± 3.8 weeks. Their results also showed that NLR had predictive value of PTB (AUC = 0.665 CI: 0.586‐0.744 sensitivity: 0.52, specificity: 0.781, PPV: 0.768, NPV: 0.538, cutoff value: 5.47). Gezer et al^39^ studied pregnant women with PTB symptoms between 34 and 37 weeks of gestation and found that NLR had predictive value for PTB with AUC:0.711 (0.662‐0.760), sensitivity: 0.651 (0.585‐0.712), specificity: 0.624 (0.548‐0.695), PPV: 0.69 (0.623‐0.751), NPV: 0.581 (0.508‐0.652), cutoff value: 6.2, LR + 1.73 (1.40‐2.14), and LR‐0.56 (0.45‐0.69).The preterm group they studied gave birth within 4.5 ± 11.1 days after CBC measurement, whereas in the present study, the interval between CBC measurement and delivery in the preterm group was 70.2 ± 15.2 days.

While the pathological mechanisms that NLR is higher in preterm delivery group compared to the term delivery group are still not fully elucidated, placental lesions consistent with maternal vascular underperfusion may be one pathway linking the rise of NLR to preterm delivery. NLR has been proved to have a significant association with systemic inflammatory response and demonstrated effective as prognostic markers in many diseases. And a study of Raffetti et al^40^ showed that NLR can also reflect the severity of the underlying systemic inflammation and provide valuable prognostic information in HIV‐infected subjects. NLR was also indicated significantly higher in malaria group than in the healthy control group.^41^ Furthermore, the dysregulation of soluble endoglin (angiogenic biomarker) was found in HIV‐infected pregnant women destined to deliver preterm.^42^ And malaria infection in early pregnancy is associated with gestational length by obstructing placental vascular development detected at delivery.^43^ Prospective studies are needed to investigate whether there are placental pathologic changes consistent with maternal vascular underperfusion, which may explain the association between NLR to preterm delivery.

In summary, previous studies have shown that NLR is of great value in predicting whether pregnant women with threatened PTB will eventually have a premature delivery. Our study further demonstrated that NLR has predictive value for PTB as early as 10 weeks before the onset of PTB symptoms.

### 4.2 PTB and MPV

MPV and PDW are two platelet volume indices that respond to platelet activation. Our study found that these two indicators, especially PDW, were significantly elevated in preterm delivery group and could be used as predictors of spontaneous PTB. Previous studies have shown that platelet activation occurs during PTB.^44^ The platelets after activation may undergo morphological changes such as the formation of pseudopods, which causes the platelet volume to expand. The platelet volume in the circulation also becomes more heterogeneous. Such changes lead to increases in MPV and PDW, especially the latter.^45^ MPV may increase in low‐grade inflammatory diseases.^46^ Chronic inflammation that occurs without a clear contagious trigger is also associated with PTB.^32^ PTB rates were significantly higher in pregnant women such as obesity^47^ and gestational diabetes.^48^ Moreover, diabetes,^49^ fatty liver disease,^50^ and obesity^49^ were found to be associated with elevated MPV. We speculate that there may be low‐grade inflammation in pregnant women with spontaneous PTB.

### 4.3 PTB and HGB

Our study also demonstrated a statistically significant increase of HGB and HCT, especially HGB in the preterm delivery group, and their high levels had predictive values for PTB. A previous study of maternal HGB and PTB risk in the Chinese population also showed that having low HGB levels in first trimester and high HGB levels in the second trimester approximately doubled the PTB risk.^51^ A possible explanation for high HGB status in the second trimester with higher PTB risk is insufficient plasma volume expansion during this period. This might lead to an increase in blood viscosity, which in turn may lead to poor blood flow in the placenta, thereby affecting the development of the fetus.^51^

A study of hemorheological adaptation during pregnancy in a Latin American population showed that HCT decreased from pre‐pregnancy levels to a minimum at around 26 weeks gestation from 10 weeks on. Blood viscosity increased during the first trimester and then decreased to a minimum at about 26 weeks of gestation.^52^ The time to evaluate CBC in pregnant women in our study was between 20‐30 weeks of gestation (mean gestational age, 24 weeks), during which blood viscosity is at a relatively low level. At this time, if the plasma volume expansion is insufficient, and the blood viscosity will rise. The blood flow in the placenta may be restricted, thus affecting the pregnancy outcome. HGB and HCT are often higher when plasma volume expansion is inadequate, which is a possible explanation for elevated HGB and HCT between 20‐30 weeks of gestation with higher risk of PTB.

### 4.4 Limitation

Our current study is retrospective, and its validity therefore very much depends on study group comparability. Although we used PSM analysis to procure comparability of some baseline characteristics between study and control groups, there may still be unknown confounding variables that were not balanced out, thus affecting the study conclusion. As the mechanism of PTB has not yet been fully elucidated and it is likely to be multifactorial, further large‐scale prospective studies are needed to confirm our findings.

## 5 CONCLUSIONS

Our study confirmed previous reports that NLR, PDW, and HGB are valuable markers to predict preterm delivery, and the diagnostic values of NLR, PDW, and HGB were superior to that of corresponding white blood cell count, platelet count, and red blood cell count. We found that the combined diagnostic marker from these three parameters could better predict preterm birth. When compared to NLR, PDW, or HGB alone, the combined diagnostic marker has the highest AUC (0.672) with 88.6% sensitivity and 40.5% specificity. To the best of our knowledge, no studies have explored the predictive value of CBC parameters in PTB as early as 10 weeks prior to preterm delivery. Current laboratory tests in routine clinical practice generally predict premature delivery by 1‐2 weeks. In addition, most of these tests use CVF as the specimens, which is known to have poor patient acceptability and compliance compared to blood sampling. Although emerging new methods hold promise in predicting PTB at earlier time, their clinical application needs to be validated by future large‐scale clinical trials. Until then, these simple and economic CBC parameters can be used as a valuable tool to predict PTB.

In summary, our study was the first to use combined diagnostic marker from NLR, PDW, and HGB to predict preterm delivery. This combined marker can supplement other markers to predict PTB about 10 weeks prior. The marker had a very high negative predictive value for PTB. Unless clinical suspicion is high, a normal combination marker value shall eliminate the need for further screening tests in an otherwise uneventful pregnancy.

## CONFLICTS OF INTEREST

The authors declare no conflict of interest.
